# Reducing Ruminal Ammonia Production With Improvement in Feed Utilization Efficiency and Performance of Murrah Buffalo (*Bubalus bubalis*) Through Dietary Supplementation of Plant-Based Feed Additive Blend

**DOI:** 10.3389/fvets.2020.00464

**Published:** 2020-08-18

**Authors:** Yendrembam Mery Chanu, Shyam Sundar Paul, Avijit Dey, Satbir Singh Dahiya

**Affiliations:** Division of Animal Nutrition and Feed Technology, ICAR-Central Institute for Research on Buffaloes, Hisar, India

**Keywords:** eucalyptus oil, licorice, nitrogen efficiency, plant bioactives, ruminal ammonia production

## Abstract

The study evaluated the potential of blends of eucalyptus oil and aqueous extract of mulethi (root of *Glycyrrhiza glabra*) to reduce rate of ruminal ammonia production without affecting feed digestion to improve nitrogen utilization efficiency and performance of Murrah buffalo (*Bubalus bubalis*). Based on preliminary independent studies with graded doses of eucalyptus oil and mulethi root aqueous extract in modulating *in vitro* rumen fermentation, four blends of feed additive comprising graded doses (5, 10, 15, and 25 μL) of eucalyptus oil and a fixed quantity (15 μL) of aqueous extract of mulethi roots were prepared and examined for their effects on *in vitro* rumen fermentation and on methane and gas production in 100-mL calibrated glass syringes by standard IVGP protocol. Rumen liquor was collected from four rumen fistulated Murrah buffaloes fed a total mixed ration. Out of four blends, blend-1 comprising 5 μL of eucalyptus oil and 15 μL of aqueous extract (233.6 g/L DW) of mulethi per 40 mL of *in vitro* medium was found to reduce ammonia production significantly (*p* < 0.001) without affecting feed digestibility. An equivalent dose of blend-1 (10.5 mL of eucalyptus oil and 7.35 g of mulethi root powder/h/day) fed to four rumen fistulated buffaloes for 24 days resulted in 50% reduction (*p* < 0.05) in rumen ammonia level with no inhibition in feed fermentation or short-chain fatty acid production. The total bacterial population including *Ruminococcus albus, Fibrobacter succinogenes, Butyrivibrio fibrisolvens*, and *Megasphaera elsdenii* as well as anaerobic fungi and methanogenic archaea remained unaffected (*p* > 0.05). Twelve buffalo calves (avg. BW 137.5 ± 9.2 kg, 8–12 months old) divided into two groups of six each and fed a total mixed ration (concentrate: roughage; 60:40) with or without supplementation of blend-1 for about 3 months demonstrated 14% increase (*p* < 0.05) in average daily gain in BW with a trend (*p* < 0.10) in improvement of feed or protein utilization efficiency (1.4 vs. 1.1 g crude protein/g average daily gain; 21.4% increase). Thus, supplementation of eucalyptus oil–mulethi root blend could reduce ruminal ammonia production and improve feed utilization efficiency in ruminants.

## Introduction

While livestock production forms one of the pillars of the global food industry, there have been huge societal concerns over the high contribution from livestock farming to atmosphere polluting gases such as methane (CH_4_), nitrous oxide (N_2_O), and ammonia (NH_3_). Methane and nitrous oxide are greenhouse gases (GHGs) that contribute to climate change, while ammonia is a potent environmental pollutant and liable for depletion of oxygen in water bodies, reduction of soil pH, and generation of harmful aerosol fine particles (PM2.5) and accompanied with an augmented threat of pulmonary diseases (1). N_2_O, the most subtle GHG, has 296 times more global warming potential than CO_2_ (2) and is also responsible for ozone depletion. Nitrogen present in animal waste is directly converted to N_2_O via microbial nitrification and denitrification, or indirectly via volatilization of ammonia gas followed by atmospheric oxidation of ammonia to N_2_O (3).

The efficiency of nitrogen utilization in ruminants is very low due to deamination of most of the amino acid nitrogen entering the rumen, resulting in a high rate of ammonia production exceeding the capacity of rumen microorganisms for utilization of ammonia (4, 5). Animal nutritionists have attempted to combat excess ruminal nitrogen degradation using naturally insoluble proteins or reducing solubility artificially by heat and formaldehyde treatments. However, a substantial increase in feed cost and potential hazards of residual formaldehyde for human health limit their application (6, 7). Monensin, an ionophore antibiotic, also reduces the ruminal degradation of amino acids (8), but inclusion of antibiotics in animal rations is worrisome. Therefore, increasing interest in use of plant bioactive compounds such as polyphenols, saponins, and essential oils in modulating rumen fermentation and protein degradation is based on their potent antimicrobial properties and safety in animal products (9). Essential oils are reported to inhibit the breakdown of proteins in the rumen, thus increasing the availability of dietary proteins to the ruminants (10, 11). Plants containing saponins have been demonstrated to shift rumen microbiome to increase efficiency of microbial protein synthesis (12, 13); however, these effects are dependent on the types of saponins as well as diets of the animals (14, 15). The major component of eucalyptus essential oils has been described as 1,8-cineole (eucalyptol), besides cryptone, α-pinene, and others (16). Mulethi (*Glycyrrhiza glabra* L.) root contains broad-spectrum bioactive compounds, i.e., flavonoids (liquiritigenin, iso-liquiritigenin), iso-flavonoids (formononetin, biochanin A), terpenoids (glycyrrhetic acid, glycyrrhizin), phenolic acids, and others, which were demonstrated to have anticancer and antioxidant properties (17, 18).

Traditionally portrayed as “black gold,” buffaloes are favorite multipurpose animals of farmers and are in fact the “bank on hooves” with a huge potential for social and economic changes for the agrarian community in Asian countries (19). Buffalo have been an integral part of livestock agriculture in Asia for more than 5,000 years, producing milk, meat, hides, and draft power. India is home to about 57% of the world's buffalo population (20) and during the past 70 years, the contribution of buffalo of +50% in the milk pool elevated India to the no. 1 world position in total milk production, while buffalo meat export earned India another distinction of being the largest beef (buffalo meat) exporting country in the world (21). Scanty *in vitro* research studies proposed reducing ammonia emission from the rumen using plant secondary metabolites (22) or by feeding dry leaves of eucalyptus (23). However, the associative effects of eucalyptus oils and mulethi (*Glycyrrhiza glabra* L.) root powder in modulating rumen fermentation and animal performance in Murrah buffalo (*Bubalus bubalis*) have not been studied. There remains a need to identify a suitable blend of feed additives that are effective in reducing the ammonia production rate in rumen without affecting feed digestion and animal performance. The present study was conducted to identify and validate a blend of plant-based feed additives that can reduce the rate of ruminal ammonia production without affecting feed digestion in rumen and thus can improve performance and feed efficiency of buffaloes.

## Materials and Methods

### Screening of Blends of Feed Additives

Four blends of feed additives comprising graded doses (5, 10, 15, and 25 μL) of eucalyptus oil and a fixed quantity (15 μL) of aqueous extract of mulethi (*Glycyrrhiza glabra* root; commonly known as licorice or sweet wood) roots were prepared and tested for their suitability to modulate *in vitro* rumen fermentation for reducing ammonia-nitrogen and methane production without impeding feed digestion. The blends were prepared based on a series of preliminary *in vitro* rumen fermentation experiments with graded doses of eucalyptus oil and aqueous extract of mulethi roots individually (24). The eucalyptus oil (80% purity) used for *in vitro* and *in vivo* rumen fermentation studies were purchased from Sigma-Aldrich Ltd. (Bangalore, India). Eucalyptus oil (60% purity) used for the *in vivo* feeding trial on growing buffaloes was purchased from Central Drug House, Delhi, India. Roots of mulethi were purchased from a local market at Hisar, Haryana, India were dried and ground to prepare extract (233.6 g/ L of distilled water) for *in vitro* studies.

### Collection of Buffalo Rumen Fluid

Rumen fluid was collected after 2 h of morning feeding from four rumen fistulated Murrah buffaloes (body weight, 700 ± 10.2 kg; age, 60 ± 3 months) fed on a roughage-based total mixed ration (wheat straw: concentrate mixture; 60:40). Four times weekly, they were offered 1 kg (DM basis) of green fodder to meet the requirements for fat-soluble vitamins. The diet was offered twice a day (10:00 and 14:30 h) by dividing into equal amounts, ensuring *ad libitum* feed intake.

### *In vitro* Rumen Fermentation

Substrate (400 mg ± 5 mg oats hay) along with each blend of additive were incubated with 40 mL of buffered rumen inoculum (buffer and strained rumen liquor in a 3:1 ratio) in calibrated glass syringes (Häberle Labortechnik, Lonsee-Ettlenschiess, Germany) of 100 mL capacity and incubated at 39°C for 24 h (25). Three syringes without substrate were incubated as blanks. The incubation procedure was repeated twice, with three replicates for every treatment.

### Estimation of Gas and Methane Production

After 24 h of incubation, the gas production was recorded by the displacement of the syringe piston. The net gas produced due to fermentation of substrate was calculated by subtracting gas produced in the blank syringes from treatments. Methane in the headspace gas was estimated as described earlier (26).

### Short-Chain Fatty Acid Estimation

After 24 h of incubation, the supernatant (1 mL) of each syringe content was taken in a microcentrifuge tube containing 0.20 mL of metaphosphoric acid (25%, v/v). The mixture was incubated for 2 h at room temperature and centrifuged at 5,000 g for 10 min. The clear supernatant was then collected and kept at −20°C for subsequent use. For analysis, 1 μL of the supernatant was injected into a gas chromatograph equipped with a flame ionization detector (FID) and glass column packed with chromosorb as described earlier (26).

### *In vitro* Dry Matter Degradability

The content of the syringes was transferred to spoutless beakers (500 mL) by repeated washings with neutral detergent solution. The contents were refluxed for 1 h and the residues were collected in preweighed sintered crucibles (G1). After drying of residues at 60 °C, neutral detergent fiber content was also estimated. True digestibility was calculated as described earlier (27).

### Estimation of Ammonia Nitrogen

For estimation of NH_3_-N concentration, the Conway disc method was utilized as described in detail earlier (26), where 1.0 mL each of supernatant from syringe contents and a saturated solution of Na_2_CO_3_ were kept in the opposite direction of outer chamber of the Conway disc and 1.0 mL of 2% boric acid solution with mixed indicators was kept in the inner chamber. The disc were capped after proper greasing and moved slowly by keeping them on the palm of the hand for proper mixing of the solutions of the outer chamber before being placed in an incubator at 39°C for 3 h. After incubation, the boric acid in the inner chamber was titrated with N/100 H_2_SO_4_ to obtain the NH_3_-N concentration. Three blanks were included for correction of NH_3_-N produced from the buffered rumen fluid in each run, and two runs were performed for each sample to obtain the representative value.

### Evaluation of the Effect of Dosing With the Best Additive Blend on *in vivo* Rumen Fermentation

Four adult rumen fistulated buffaloes were fed a roughage-based diet (concentrate level 40% and wheat straw 60%). Animals were dosed with the best blend (blend-1) of additives for 24 days. In the *in vitro* trial, a satisfactory result was found with blend-1, i.e., a mixture of 5 μL of eucalyptus oil with 15 μL of mulethi root extract (5.84 g of mulethi/25 mL DW) per 40 mL of *in vitro* incubation fluid. This dose was utilized for calculating the dose in fistulated buffaloes of 700 kg BW assuming rumen volume of 10% of BW and 20% higher than the calculated dose to take care of calculation error and the dynamic system. Based on these calculations fistulated buffaloes were fed with a blend of 10.5 mL of eucalyptus oil of 80% purity and 7.35 g of mulethi powder daily during the dosing period. The eucalyptus–mulethi blend (EMB) was mixed with a portion of concentrate mixture and offered at 10:00 h daily. The animals consumed the entire concentrate mixture containing the feed additive within 15–20 min of offering. Sampling of rumen content was done over 3 consecutive days after 21 days post-dosing. Ammonia and short-chain fatty acid (SCFA) levels in rumen fluid were assayed as per the procedure described earlier.

### Estimation of Ruminal Enzymes

Rumen liquor was centrifuged at 12,000 *g* at 4°C for 15 min and the supernatant was utilized for estimation of enzymes. Activities of carboxymethyl cellulase (endo-1,4 β-glucanase), xylanase (endo-1,4 β-xylanase), β-glucosidase (β-d-glucoside glucohydrolase), and acetyl esterase (acetic-ester acetylhydrolase) activities were estimated as described earlier (27). Protease activity was measured with azocasein (Sigma Chemical Co., St. Louis, MO, USA) as substrate as described earlier (28). The protein content of the enzyme sample was estimated as per Lowry et al. (29).

### Chemical Analysis of Samples

Feed samples were analyzed for dry matter (930.15), ash (942.05), crude protein (988.05), and fat (920.39) by the Association of Official Analytical Chemistry (AOAC) procedures (30) and fiber fractions (neutral detergent fiber [NDF] and acid detergent fibre [ADF]) according to Van Soest et al. (31). NDF was analyzed without decalin, sodium sulfite, and α-amylase and expressed with residual ash.

### Extraction of DNA and qPCR-Based Quantification of Different Rumen Microbial Groups

Metagenomic DNA was extracted from the rumen content of each fistulated buffalo (32). The DNA quality was evaluated using agarose gel (1%) electrophoresis, and DNA yield was quantified using a nano-drop spectrophotometer (Invitrogen Corporation, Carlsbad, CA, USA). The DNA samples were stored at −20°C until analysis. The abundances of total archaea and select bacterial species were quantified using SYBR Green-based quantitative real time-PCR (qPCR) using an Applied Biosystem (StepOnePlus) real-time PCR machine (33). To reduce variations, the qPCR assay for each species or group of microbes was performed in triplicate for both the standards and the metagenomic DNA samples using the same master mix and the same PCR plate. The 10-μL master mix contains 5 μL of Power SYBR Green master mix (Invitrogen Singapore Pte. Ltd., Singapore), 1 μL of forward primer, 1 μL of reverse primer, 1 μL of template DNA, and 2 μL of nuclease-free water. The real-time qPCR primers used in this study are listed in Table 1. Cycling parameters for qPCR consisted of initial denaturation at 95°C for 10 min for 1 cycle, 40 cycles of amplification (denaturation at 95°C for 15 s, annealing at annealing temperature of the respective primer pairs for 10 s, and elongation at 60°C for 50 s with data acquisition at the 60°C step) and melting curve (95°C for 2 min, 60°C for 15 s, and then increasing temperature at the rate of 0.2°C every 5 s up to 95°C and measuring the fluorescence of the melting amplicon at 5-s intervals). Nonspecific primer amplification was assessed by the inclusion of no template controls. qPCR products were checked though agarose gel electrophoresis for the presence of a single band/product. The absolute abundances were expressed as *rrs* gene copies/mL of sample.

**Table 1:** Primers used for real-time qPCR.

**Primer name**	**Sequence (5^**′**^ → 3^**′**^)**	**Expected amplicon size (bp)**	**Annealing temperature (^**°**^C)**	**References**
Total bacteria	F-CCTACGGGAGGCAGCAG R-ATTACCGCGGCTGCTGG	194	60	Singh et al. (34)
*Ruminococcus albus*	F-CCCTAAAAGCAGTCTTAGTTCG R-CCTCCTTGCGGTTAGAACA	175	55	Singh et al. (34)
Total archaea	F-GGATTAGATACCCSGGTAGT R-GTTGARTCCAATTAAACCGCA	173	56	Hook et al. (35)
Total anaerobic fungi	F-GAGGAAGTAAAAGTCGTAACAAGGTTTC R-CAAATTCACAAAGGGTAGGATGATT	120	56	Lwin et al. (36)
*Butyrivibrio fibrisolvens*	F-ACCGCATAAGCGCACGGA R-CGGGTCCATCTTGTACCGATAAAT	65	62	Stevenson and Weimer (37)
*Megasphaera elsdenii*	F-GACCGAAACTGCGATGCTAGA R-CGCCTCAGCGTCAGTTGTC	130	57.8	Bekele et al. (38)

### Evaluation of the Best Blend of Additives (EMB) on Performance of Growing Buffaloes

**Figure 1:**
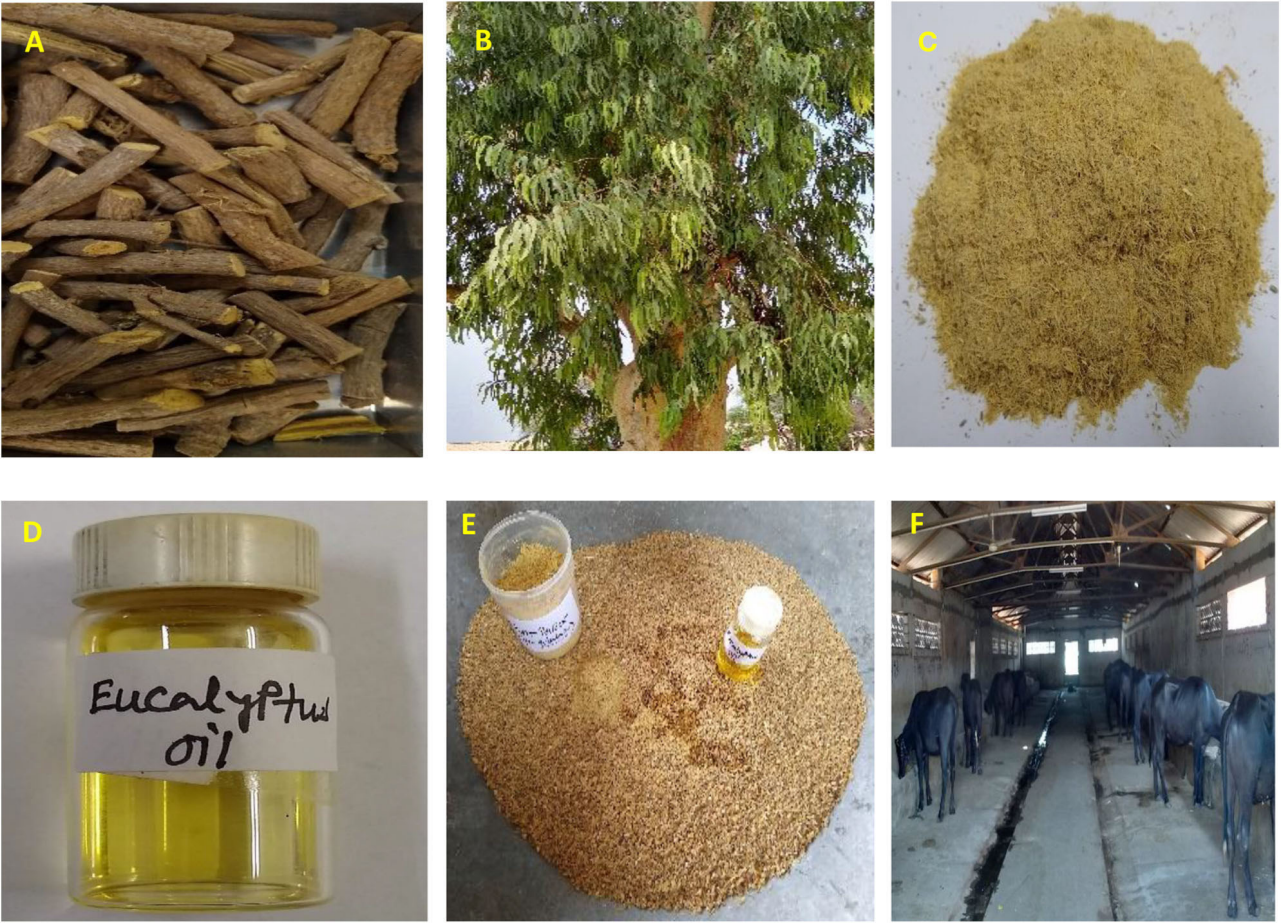
Preparation and supplementation of eucalyptus oil–mulethi powder blend to buffalo calves. **(A)** Mulethi roots. **(B)** Eucalyptus tree. **(C)** Grounded mulethi roots by Willy grinder. **(D)** Eucalyptus oil. **(E)** Requisite quantities of Mulethi root powder and eucalyptus oil were placed into a small portion of concentrate mixture and mixed uniformly. **(F)** The premix was placed into the manger and mixed with the other portion of concentrate mixture and fed to buffalo calves.

Twelve growing buffalo calves (avg. BW 137.5 ± 9.2 kg, 8–12 months old) were selected from an animal farm and assigned randomly to two groups of six each. All the experimental calves were fed a diet containing wheat straw and concentrate mixture (crude protein [CP] 20.04%) in accordance with the nutritional requirement for maintenance and growth (39). One group (control without any additives [CON]) received a control diet (40:60 roughage:concentrate) and the other group (EMB) received the control diet along with feed additive containing eucalyptus oil–mulethi blend (EMB). Each buffalo calf in the EMB group was fed a blend of 2 mL of eucalyptus oil (60% purity) and 1.05 g of mulethi root powder per 100 kg BW by mixing with a small portion of concentrate mixture (Figure 1) offered at 10:00 h daily. The animals consumed the concentrate mixture containing additive within 15–20 min of offering. The feeding trial was continued for ~3 months (84 days) and one digestibility trial of 6 days was performed at the mid-phase of the feeding trial to assess digestibility of nutrients by total collection of feces. Parameters studied were growth rate, feed intake, and digestibility of nutrients. Blood samples were collected from the jugular vein of each experimental calf at 0, 30, 60, and 84 days of feeding trial for estimation of blood biochemical parameters (alanine aminotransferase [ALT], aspartate aminotransferase [AST], total proteion, albumin, globulin, triglyceride, cholesterol, and urea) with commercial biochemical assay kits (Coral Clinical Systems, Verna, Goa, India) on an automated biochemical analyzer (Coralyzer200, Tulip Diagnostics, Goa, India).

### Statistical Analysis

All statistical analyses were performed using SPSS 17.0 (40). All the data from *in vitro* experiments were analyzed by one-way ANOVA in a completely randomized design and the differences between the means were compared by Tuckey's test. Data from *in vivo* studies were analyzed by a *t*-test. The log-transformation of the abundance (*rrs* gene copy numbers/30 ng of DNA) of ruminal microorganisms was done before statistical analysis for improved normality. Differences in mean values were considered significant if *p* ≤ 0.05, and trends toward significance were examined at 0.05 < *p* < 0.10.

## Results

Although all four blends examined in the present study were observed to reduce (*p* < 0.001) the ammonia-N and methane production under the *in vitro* fermentation system, only blend-1 containing the lowest dose of eucalyptus oil (5 μL) and 15 μL of aqueous extract of mulethi roots was found to reduce these parameters without impeding true digestibility of dry matter (TDDM) and SCFA production (Table 2). Therefore, blend-1 was found to be best among the others.

**Table 2:** Effects of adding various blends of eucalyptus oil and aqueous extracts of mulethi roots to rumen fluid of buffalo on *in vitro* rumen fermentation and methane and total gas production.

**Attributes**	**NH_**3**_-N (mg/dL)**	**TDDM (%)**	**Total gas (mL)**	**Methane (%)**	**Acetate (mM/dL)**	**Propionate (mM/dL)**	**Butyrate (mM/dL)**	**A:P**
CON	51.3^c^	79.8^b^	45.0^d^	18.7^d^	14.0^b^	3.6^b^	1.7	3.9^b^
Blend-1	43.8^b^	78.2^b^	43.0^c^	17.7^c^	12.3^b^	3.1^b^	1.1	3.9^b^
Blend-2	43.4^b^	75.8^a^	41.5^b, c^	16.5^b, c^	7.6^a^	1.9^a^	0.98	3.9^b^
Blend-3	42.9^a, b^	74.9^a^	40.5^a, b^	16.3^b^	7.9^a^	1.9^a^	1.1	4.1^b^
Blend-4	40.6^a^	74.1^a^	39.0^a^	13.5^a^	7.4^a^	1.7^a^	1.0	4.3^a^
SEM	1.15	0.89	1.06	0.793	1.25	0.65	0.45	0.27
*p*-value	<0.001	<0.001	0.001	0.001	0.007	0.003	0.23	0.026

*Blend-1, eucalyptus oil (5 μL) + mulethi extract (15 μL); Blend-2, eucalyptus oil (10 μL) + mulethi extract (15 μL); Blend-3, eucalyptus oil (15 μL) + mulethi extract (15 μL); Blend-4, eucalyptus oil (25 μL) + mulethi extract (15 μL); CON, control without any additives. A:P, acetate: propionate; NH_3_-N, ammonia nitrogen; SEM, Standard error of mean; TDDM, true digestibility of dry matter. Means followed by different superscript letters (a–d) in a column differ significantly (p < 0.01)*.

### Effect of the Best Additive Blend (Blend-1) on *in vivo* Rumen Fermentation, Rumen Fibrolytic Enzymes, and Microbial Population Density

There was an ~50% reduction (*p* < 0.01) in ammonia-N concentration in the rumen fluid of buffaloes supplemented with EMB in samples of 2 h post feeding (Table 3). Although individual SCFA production remained comparable (*p* > 0.05), the ratio of acetate to propionate increased (*p* < 0.05) in EMB-supplemented buffaloes compared to the CON. Although the ruminal enzymes, i.e., carboxymethyl cellulase (CMCase), acetyl esterase, and xylanase levels were not affected (*p* > 0.05) by the supplementation of additive (Table 3), there were a trend (*p* < 0.10) in increasing CMCase and xylanase activities. However, the protease and β-glucosidase levels were lower (*p* < 0.01) in the EMB group compared to CON. The real-time PCR data (Table 3) indicated that there were no changes (*p* > 0.05) in number of total bacteria, fibrolytic bacteria, or *Megasphaera elsdenii* and total methanogenic archaea in the supplemented group compared with CON. A trend (*p* < 0.10) in reducing anaerobic fungal population was noted.

**Table 3:** Effects of best blend of feed additives (blend-1) supplementation to rumen cannulated buffaloes on ruminal pH, short-chain fatty acids, ammonia-N, enzyme activities, and abundances of microbial population.

**Attributes**	**CON**	**EMB**	**SEM**	***p***
pH	6.4	6.5	0.12	0.776
**SHORT-CHAIN FATTY ACID PRODUCTION (mM/dL)**				
Acetate	10.2	7.9	1.00	0.183
Propionate	2.9	1.9	0.38	0.114
Butyrate	1.4	0.81	0.23	0.122
Acetate: propionate	3.5^a^	4.1^b^	0.12	0.023
NH_3_-N (mg/dL)^*^	22.2^b^	11.3^a^	1.59	0.008
**ENZYME ACTIVITIES (mIU/mL)**				
Carboxymethyl cellulase (endo-1,4 β-glucanase)	19.3	24.6	1.83	0.060
Xylanase (endo-1,4 β-xylanase)	84.9	73.7	4.12	0.074
β-Glucosidase (β-d-Glucoside glucohydrolase)	16.7^b^	11.7^a^	0.76	0.0001
Acetyl esterase (acetic-ester acetylhydrolase)	38.2	39.3	1.14	0.489
Protease	18.2^b^	7.9^a^	0.020	0.0001
**MICROBIAL ABUNDANCE (log16 S RDNA copy numbers/30 ng of DNA)**				
Total bacteria	11.85	11.35	0.520	0.536
*Ruminococcus albus*	6.76	5.92	0.315	0.132
*Fibrobacter succinogenes*	7.66	6.81	0.900	0.542
*Butyrivibrio fibrisolvens*	6.32	6.21	0.225	0.379
*Megasphaera elsdenii*	4.48	4.83	0.345	0.142
*Anaerobic fungi*	8.03	7.10	0.256	0.062
Total archaea	7.41	6.34	0.402	0.120

*CON, control group; EMB, eucalyptus–mulethi blend (control diet with a blend of 10.5 mL of eucalyptus oil and 7.35 g of mulethi root powder/h/day); SEM, standard error of mean*.

* *Measured at 2 h post-feeding. Means followed by different superscript letters (a, b) in a row differ significantly (p < 0.05)*.

### Effect of Feeding the Additive Blend on Growing Buffaloes

The comparable (*p* > 0.05) intake of wheat straw or concentrate mixture between the CON and supplemented groups (Table 4) indicated that the additive mixture did not affect feed intake or palatability of feeds.

**Table 4:** Growth rate and feed efficiency of buffalo calves supplemented with feed additive.

**Attributes**	**CON**	**EMB**	**SEM**	***p***
**BODY WEIGHT GAIN**				
Total gain^*^ (kg)	60.8^a^	69.4^b^	3.06	0.049
ADG (g)	724.0^a^	826.0^b^	37.5	0.049
**DRY MATTER INTAKE (kg/day)**				
Concentrate	3.13	3.19	0.19	0.444
Wheat straw	1.45	1.49	0.11	0.25
Total	4.59	4.69	0.29	0.304
FCR	6.37	5.73	0.38	0.057
CP intake (g/day)/g ADG	1.40	1.10	0.11	0.084
TDN intake (g/day)/g ADG	5.14	4.11	0.45	0.115

*CON (control group), diet containing a concentrate mixture and wheat straw EMB; (treatment group) = control diet supplemented with a blend of 2 mL of eucalyptus oil (60% purity) and 1.05 g of mulethi root powder per 100 kg BW per day. ADG, average daily gain; CP, crude protein; FCR, feed conversion ratio (kg dry matter intake/kg gain); SEM, standard error of mean; TDN, total digestible nutrients*.

* *After 84 days*.

a, b *Mean values bearing different superscripts within a row vary significantly (p < 0.05)*.

The total gain (kg) in body weight at the end of the feeding trial and average daily gain (ADG, g) was higher (*p* < 0.05) in buffalo calves in the EMB group compared to CON (Table 4). The feed conversion ratio (FCR) and efficiency of conversion of dietary protein for body weight gain tended (*p* < 0.10) to be better in the supplemented group compared to CON.

The digestibility coefficient of various nutrients (Table 5) did not differ (*p* > 0.05) in animals in both the CON and supplemented groups, indicating that the feed additive did not exert any adverse effect on whole gastrointestinal tract digestibility of nutrients in calves (Table 5). The blood biochemical parameters (Table 6) in animals of both groups remained comparable (*p* > 0.05) throughout the experimental feeding, although blood urea level tended (*p* < 0.10) to be reduced at the 84th day in EMB-supplemented buffaloes compared to CON.

**Table 5:** Nutrient digestibility and nutritive value of diets fed to buffalo calves supplemented with feed additives.

**Attributes**	**CON**	**EMB**	**SEM**	***p***
**NUTRIENT DIGESTIBILITY (%)**				
Dry matter	58.9	59.7	1.40	0.701
Organic matter	59.3	60.3	1.54	0.920
Crude protein	72.2	73.8	1.26	0.378
Ether extract	71.5	75.0	2.08	0.261
Neutral detergent fiber	56.8	58.9	1.55	0.353
Acid detergent fiber	52.3	53.7	1.51	0.503
**NUTRIENT DENSITY (%)**				
CP	15.7	15.6	0.39	0.793
DCP	11.4	11.4	0.43	0.843
TDN	57.2	58.0	1.24	0.639

*CON, control group; CP, crude protein; DCP, digestible crude protein; EMB, eucalyptus–Mulethi blend supplemented group; SEM, standard error of mean. TDN, total digestible nutrients*.

**Table 6:** Effects of supplementation of the feed additives on blood biochemical profile of buffalo calves.

**Parameters**	**Days**	**CON**	**EMB**	**SEM**	***p***
Urea (mg/dL)	0 day	18.00	15.60	3.21	0.610
	30 day	30.06	27.38	7.42	0.804
	60 day	33.52	32.82	3.87	0.901
	84 day	43.22	30.22	4.51	0.087
Cholesterol (mg/dL)	0 day	70.20	72.88	3.68	0.620
	30 day	82.85	81.55	4.14	0.828
	60 day	86.84	85.9	2.09	0.758
	84 day	82.58	78.22	2.9	0.311
Triglyceride (mg/dL)	0 day	66.78	65.06	4.12	0.775
	30 day	49.38	45.24	3.44	0.419
	60 day	68.2	71.35	4.58	0.338
	84 day	53.3	59.06	5.37	0.470
Total protein (g/dL)	0 day	5.88	5.8	0.206	0.781
	30 day	5.58	5.45	0.116	0.434
	60 day	5.68	5.56	0.181	0.658
	84 day	5.83	5.61	0.183	0.421
Albumin (g/dL)	0 day	0.98	0.98	0.025	1.00
	30 day	0.91	0.85	0.131	0.282
	60 day	0.98	0.98	0.025	1.00
	84 day	1.05	1.03	0.034	0.734
Globulin (g/dL)	0 day	4.9	4.82	0.179	0.749
	30 day	4.66	4.6	0.112	0.683
	60 day	4.7	4.58	0.169	0.637
	84 day	4.78	4.58	0.176	0.440
AST (units/L)	0 day	171.57	178.75	10.16	0.641
	30 day	132.85	131.45	13.01	0.941
	60 day	117.08	125.08	4.51	0.297
	84 day	123.00	118.85	3.99	0.479
ALT (units/L)	0 day	79.92	80.48	4.58	0.933
	30 day	57.52	50.56	2.47	0.081
	60 day	66.12	65.7	2.59	0.912
	84 day	65.46	62.38	2.2	0.353

*ALT, alanine aminotransferase; AST, aspartate aminotransferase; CON, control group; EMB, eucalyptus–Mulethi blend supplemented group; SEM, standard error of mean*.

## Discussion

Feed additives of plant origin are gaining importance in the diet of ruminants for their antimicrobial properties to modulate rumen fermentation to increase animal production and reduce environmental pollution. The activity of essential oils including eucalyptus oil depends on the presence of the kinds of functional groups and their proportions. The major secondary metabolite present in eucalyptus oil is 1,8-cineole, which is responsible for the antimicrobial property (41). The inhibition of ruminal hyper-ammonia-producing bacteria (HAB) responsible for deamination of amino acids to ammonia in the protein degradation pathway by eucalyptus essential oils (EOs) could be the reason for lowered ammonia production (42). The reduction of ammonia-N and methane production on *in vitro* incubation of oats hay with eucalyptus oil was also reported by Singh et al. (26). However, in *in vivo* studies involving other EOs, many researchers (43–45) demonstrated similar concentration of NH_3_-N in rumen fluid of both control and EO-supplemented animals. A lower (*p* < 0.05) level of NH_3_-N was reported by Patra (46) in garlic-fed fistulated buffaloes. While studying the effects of some EOs and their main active compounds on rumen fermentation, Busquet et al. (47) demonstrated marked inhibition of NH_3_-N concentration at high doses with marginal to no effects at moderate and low doses, respectively. Earlier, Sallam et al. (23) reported a reduction in ruminal ammonia nitrogen concentration on feeding fresh and dry leaves of eucalyptus as compared to alfalfa hay.

Mulethi is known to contain tannins and saponins besides other plant bioactive compounds (48). A substantial reduction of ruminal proteolytic activity as well as ammonia nitrogen concentration in sheep fed condensed tannin (CT) from *Lotus corniculatus* was demonstrated by Min et al. (49). Similarly, a reduced ruminal ammonia nitrogen concentration by supplementation of *Yucca schidigera* (rich in saponin) extract was described in a continuous incubation system (50). As proposed previously (42, 51) for other EOs, the reduction in ammonia nitrogen production by the additive blends used in the present study could be attributed to direct inhibition of HAB, which are low in number but produce ammonia at a much faster rate than other rumen microbes. The current observation that high doses of eucalyptus oils in blends-2,−3, and−4 tested tend to reduce *in vitro* dry matter digestibility, total gas and methane production, and ruminal SCFA production at higher doses but the lower dose (blend-1) may not affect SCFA production is consistent with those of Castillejos et al. (52) and Patra and Yu (53).

The lower (*p* < 0.01) protease activity observed in this study is expected to reduce amino acid degradation in rumen, which in turn will increase the supply of proteins for animals. McInotsh et al. (10) also observed inhibition of some HAB growth with addition of a mixture of EOs. As there was a severe decrease in ammonia concentration in rumen fluid on feeding the additive, it is possible that the additive might have selectively inhibited some of the HAB, including some of the uncultured group, without affecting total bacterial count. In the present study, the methanogenic archaea population tended to decline in the treatment group, which indicated that the identified additive can have an added advantage by reducing methane emission in addition to lower ammonia emission. The increasing (*p* < 0.10) activities of CMCase and xylanase might have contributed toward digestion of feed in the animals, resulting in greater acetate production compared to propionate, thus increasing the acetate to propionate ratio. The quantity and species of HAB varies with the diet. Again the effects of EOs in the stationary phase under *in vitro* conditions and dynamic system of rumen may be different. This may explain the higher reduction of ammonia-N in *in vivo* experiments (10). The trend in reducing rumen anaerobic fungi in EMB-supplemented buffaloes is due to the direct inhibitory effect of EOs on these microbes (54).

In the current study, no adverse effect of the additive on feed intake was observed. In contrast, reduction in feed intake on feeding some of the EOs was reported in other studies that might be related to palatability problems (55). Scant information on the effect of feeding blend of eucalyptus oil plus mulethi on whole gastrointestinal tract digestibility of nutrients is available for comparison. Benchaar et al. (56) and Santos et al. (57) reported similar nutrient digestibility in dairy cows fed (2 g/day) with a mixture of EOs, whereas, higher (13%) dry matter (DM) digestibility was described by Yang et al. (45) in dairy cows supplemented (2 g/day) with juniper berry EOs.

Data on the effect of a feeding blend of eucalyptus oil and mulethi on growth rate are not available for comparison. Although feed intake and nutrient digestibility remained comparable for both the groups, better utilization of nitrogen perhaps increased the ADG in EMB-supplemented calves, which was also evidenced by better FCR (*p* = 0.057) and protein utilization efficiency (1.4 vs. 1.1 g CPI/g ADG; 21.4% increase). In an experiment on beef cattle supplemented (2 or 4 g/day) with a mixture of EOs, Benchaar et al. (56) demonstrated comparable ADG in both control and supplemented groups. However, higher ADG was reported by Chaves et al. (58) on supplementation (0.2 g/kg of DM) of either cinnamaldehyde or juniper berry EOs in growing lambs. The present results corroborate the description of Khateri et al. (59), in which feeding of a mixture of EOs in ruminants had no effect on blood biochemical parameters; however, a trend to reduce blood urea concentration at a later stage of the present feeding experiment in EMB-supplemented buffaloes could also explain the reduced degradation of protein in rumen.

## Conclusions

The blend of a low dose of eucalyptus oil plus mulethi root extract was found to have a potential to reduce the rate of ruminal ammonia production without affecting feed digestion and thus improving feed efficiency. A dose of 10.5 mL of eucalyptus oil of 80% purity and 7.35 g of mulethi root powder daily can reduce ruminal ammonia production by 50% in adult buffaloes weighing 700 kg without affecting feed digestibility. *In vivo* studies in growing buffaloes confirmed that this feed additive can improve growth performance and tend to improve protein efficiency and feed utilization efficiency in buffaloes.

## Data Availability Statement

The raw data supporting the conclusions of this article will be made available by the authors, without undue reservation.

## Ethics Statement

Experimental protocol was approved by the ICAR-CIRB animal ethics committee (IEAC protocol no. 1/2016).

## Author Contributions

YC and SP: conceived and designed the experiment. YC, SP, and AD: collected raw materials and performed the experiment. AD and SD: analyzed the data and interpreted the results. SP and AD: wrote the manuscript. All authors contributed to the article and approved the submitted version.

## Conflict of Interest

The authors declare that the research was conducted in the absence of any commercial or financial relationships that could be construed as a potential conflict of interest.
