# Supplementary material for: Reducing Ruminal Ammonia Production With Improvement in Feed Utilization Efficiency and Performance of Murrah Buffalo (Bubalus bubalis) Through Dietary Supplementation of Plant-Based Feed Additive Blend
Source: Front Vet Sci. 2020 Aug 18;7:464. doi: 10.3389/fvets.2020.00464 (PMC7461841; doi:10.3389/fvets.2020.00464)
Supplement: Supplementary file 1 [file Table_1.DOCX]

**Additional Tables**

**Table 1. Ingredients and chemical composition of the experimental diet**

| **Constituents** | **Concentrate mixture** | **Wheat straw** |
| --- | --- | --- |
| ***Ingredients (%)*** | | |
| Maize | 30 | - |
| Wheat bran | 32 | - |
| Groundnut cake | 15 | - |
| Mustard cake | 20 | - |
| Mineral mixture | 2 | - |
| Common salt | 1 | - |
| ***Chemical composition (% DM)*** | | |
| Organic Matter (OM) | 88.2 | 87.2 |
| Crude Protein (CP) | 20.04 | 2.98 |
| Ether Extract (EE) | 4.08 | 0.59 |
| Total ash | 11.8 | 12.8 |
| Natural Detergent Fiber (NDF) | 18.83 | 73.2 |
| Acid Detergent Fiber (ADF) | 10.83 | 51.22 |
